# New Perspectives on Roles of Alpha-Synuclein in Parkinson’s Disease

**DOI:** 10.3389/fnagi.2018.00370

**Published:** 2018-11-22

**Authors:** Guoxin Zhang, Yun Xia, Fang Wan, Kai Ma, Xingfang Guo, Liang Kou, Sijia Yin, Chao Han, Ling Liu, Jinsha Huang, Nian Xiong, Tao Wang

**Affiliations:** ^1^Department of Neurology, Union Hospital, Tongji Medical College, Huazhong University of Science and Technology, Wuhan, China; ^2^Department of Neurology, Anhui Provincial Hospital, The First Affiliated Hospital of Science and Technology of China, Hefei, China

**Keywords:** alpha-synuclein, Parkinson’s disease, prion-like, neurodegeneration, neurotherapy

## Abstract

Parkinson’s disease (PD) is one of the synucleinopathies spectrum of disorders typified by the presence of intraneuronal protein inclusions. It is primarily composed of misfolded and aggregated forms of alpha-synuclein (α-syn), the toxicity of which has been attributed to the transition from an α-helical conformation to a β-sheetrich structure that polymerizes to form toxic oligomers. This could spread and initiate the formation of “LB-like aggregates,” by transcellular mechanisms with seeding and subsequent permissive templating. This hypothesis postulates that α-syn is a prion-like pathological agent and responsible for the progression of Parkinson’s pathology. Moreover, the involvement of the inflammatory response in PD pathogenesis has been reported on the excessive microglial activation and production of pro-inflammatory cytokines. At last, we describe several treatment approaches that target the pathogenic α-syn protein, especially the oligomers, which are currently being tested in advanced animal experiments or are already in clinical trials. However, there are current challenges with therapies that target α-syn, for example, difficulties in identifying varying α-syn conformations within different individuals as well as both the cost and need of long-duration large trials.

## Introduction

Parkinson’s disease (PD) is characterized by the accumulation of misfolded fibrillar alpha-synuclein (α-syn) that significantly features Lewy bodies and Lewy neurites (LBs/LNs). Investigations into protein aggregation have revealed a propensity of monomeric α-syn to form small soluble aggregates such as oligomers and large insoluble fibrillar bodies. Certain α-syn oligomeric species, conferring toxicity to neurons, were reported to play a key role in the neurodegenerative process, and different mechanisms are involved (see below). It is well known that genetic mutations of familial PD have a crucial role in α-syn toxicity. Alpha-synuclein accumulation is a known cause of underlying the pathophysiology of PD. By contrast, sporadic forms of PD associated with genes other than those encoding α-syn also result in the α-syn accumulation. This suggests that mutations in these genes might exacerbate the role of upstream factors such as oxidation, nitration, and decreased proteasomal and lysosomal functions. Glial neuro-inflammation, as well as Golgi trafficking and calcium buffering, might lead to α-syn accumulation through an independent pathway specific to that gene. In addition, emerging evidence suggests that α-syn spreads from neuron to neuron or glia via self-amplification and propagates in a stereotypical and topographical pattern among neighboring cells (Dehay et al., 2016). According to the distribution of LBs in postmortem brains of patients with PD, researchers saw a gradually expanding distribution of Lewy-like deposits, following the anatomical pathways from the olfactory bulb to the substantia nigra pars compacta (SNpc) and finally the cortex (Braak et al., 2003). The mode of spreading from peripheral tissues such as olfactory bulb to the brain seems to explain the conjecture that a prion-like fashion could be responsible for the staged propagation (Rey et al., 2016). Furthermore, recent research into the disequilibrium and the complexity of microglial reaction has become a focal point. As the major scavenger for extracellular α-syn aggregates, an excessive microglial activation increases the production of proinflammatory cytokines including tumor necrosis factor alpha (TNF-α), interleukin-1-β (IL-1β), interleukin-6 (IL-6), and interferon-γ (INF-γ). Finally, current treatments for PD are focused merely on symptom-controling, limited by lack of disease-modifying therapeutic potentials aimed at reducing α-syn toxicity. The α-syn-directed therapeutics emphasized on alleviating the neurotoxic gain of α-syn aggregation by establishing a new balance in α-syn synthesis, aggregation, and clearance (Dehay et al., 2015). The development of immunotherapeutic approaches targeting α-syn, especially the oligomeric species, has received considerable attention and it is promising. In this review, we focus on the structure, toxicity, and the potential role of α-syn oligomers as mediators in prion-like hypothesis, microglia-mediated inflammation, and new areas of therapeutic investigation for PD.

## Structure and Toxicity

### Structure of α-Syn

Primarily as a natively unfolded cytosolic protein, α-syn, an abundant 14-kDa protein consisting of 140 amino acids, is comprised of 3 domains: (1) an N-terminal lipid-binding α-helix, (2) a non-amyloid–β component (NAC) domain, and (3) an unstructured C-terminus. The N-terminal, characterized by seven 11 amino acid repeats, plays a role in binding to membranes, upon which it adopts an α-helical secondary structure and further misfolds into aggregates (Eliezer et al., 2001). When this occurs, a random coil of the NAC region, a highly hydrophobic sequence underlying the aggregate nature, forms β-sheets and leads to protofibrils and fibrils. The unstructured C-terminus contains a large number of charged residues, that contribute to inhibiting this fibril formation, and is home to significant post-translational modification (Hasegawa et al., 2002). Negative stain electron microscopy (EM) images revealed α-syn fibril polymorphs with different widths, and most of these were 10-nm wide straight or twisted filaments, while few of these were 5-nm wide straight filaments (Spillantini et al., 1998a,b). Atomic insights into the α-syn fibril architecture have also been presented in many structural studies. Using micro-electron diffraction (microED) (Rodriguez et al., 2015) to analyze the structures of the preNAC region, NACore regions (Non-Amyloid-β Component core), and amyloidogenic segments, a pair of tightly mated in-register β-sheets forming a steric zipper was found. Moreover, a solid-state nuclear magnetic resonance (ssNMR) structure of recombinant α-syn confirmed a Greek-key β-sheet motif in the hydrophobic core of a single fibril filament (Tuttle et al., 2016), where salt bridges (E46-K80), a glutamine ladder (Q79), and hydrophobic packing of aromatic residues (F94) contribute to the stability of the in-register β-sheets. However, a recent study on the fibrillar structure, using cryo-electron microscopy (cryo-EM), revealed a different α-syn fold, where the fibrillar core is constituted by two back-to-back protofilaments (Guerrero-Ferreira et al., 2018). Two recent studies were published in regard to amyloid fibril structure of full-length α-syn using electron cryomicroscopy (cryo-EM) helical reconstruction. Li B. et al. (2018) present the three-dimensional (3D) structures of the two predominant species of α-syn, a rod and a twister, both at 3.7 Å resolution. The atomic models reveal that both polymorphs share a kernel structure of a bent β-arch, but differ in their inter-protofilament interfaces (Li B. et al., 2018). Another study using cryo-EM at an overall resolution of 3.07 Å to investigate an amyloid fibril structure of full-length α-syn showed two protofilaments intertwining along an approximate 21-screw axis into a left-handed helix, each of which features a Greek key-like topology (Li Y. et al., 2018). Recently, research has also identified specific structural elements of α-syn oligomers that give rise to cellular toxicity by disrupting the integrity of biological membranes. These include an exposed, highly lipophilic region of oligomeric α-syn that promote strong interactions with the membrane surface and a rigid oligomeric core that is rich in β-sheet structure, which can enter the lipid bilayer (Fusco et al., 2017). In addition, the effects of pathological α-syn might include the alteration of mitochondrial function and the behavior of Golgi apparatus, synaptic dysfunction, and nuclear dysfunction (Wong and Krainc, 2017). However, the direct link between α-syn and PD pathology, including death of dopaminergic neurons, is not entirely clear, with some even suggesting that α-syn pathology in the form of LBs may be neuroprotective, in response to increased levels of misfolded proteins and to facilitate the clearance of these potentially toxic proteins.

### Native and Toxic Conformations of α-Syn

Interest in the toxicity of α-syn began when mutations in the SNCA gene encoding the protein were identified in cases of familial PD. Missense mutant cases of PD appear to have an earlier age of onset than sporadic cases of PD and involve a faster rate of motor decline. All of the missense mutations (A53T, A30P, E46K, H50Q, and G51D, and the newly described A18T, pA29S) identified to date are notable for being confined to two helix-forming regions of the N-terminal domain (Hoffman-Zacharska et al., 2013). This has highlighted their destabilizing effect on the N-terminal region and increased the propensity for forming β-structure, which may promote aggregation. Additionally, gene duplications and triplications of SNCA can cause familial PD in an α-syn dose-dependent manner (Chartier-Harlin et al., 2004). Patients with a gene dosage of ∼1.5, or three copies of SNCA, have a disease presentation similar to that of late-onset sporadic PD, while patients with a gene dosage of ∼2.0, or four copies of SNCA, tend to develop severe early-onset PD with extensive dementia and non-motor features. Furthermore, genome-wide association studies have shown a correlation between polymorphisms in the SNCA gene and an increased risk of PD, including in the microsatellite “Rep1” variant in the promoter region of SNCA (Maraganore et al., 2006). Lastly, epigenetic changes can also enhance SNCA expression, including reduced CpG methylation of the SNCA intron 1 observed in brains of patients with sporadic PD (Jowaed et al., 2010).

**FIGURE 1:**
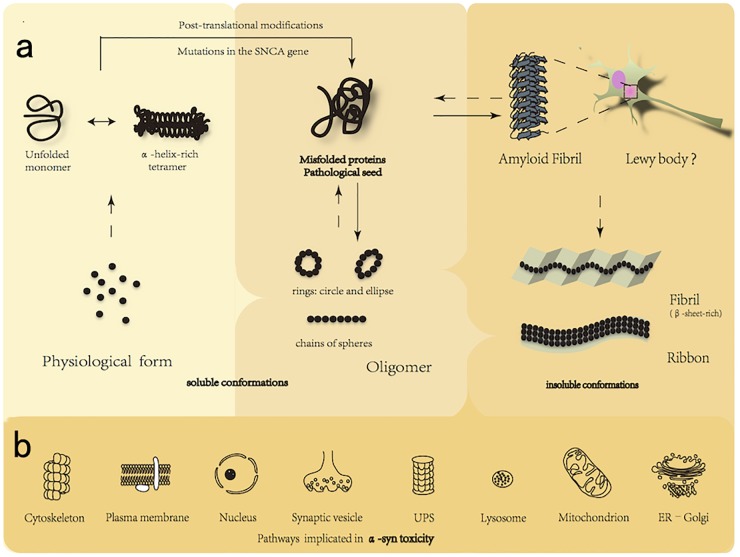
**(a)** Native and toxic conformations of α-syn. Alpha-synuclein is able to transform into multiple different conformations, including monomers (predominant in a α-helical confirmation), tetramers, higher-level oligomers (soluble conformations), and fibrils (highly ordered insoluble conformations characterized by β-sheet conformation). Alpha-synuclein exists in a native conformation as monomers as well in a dynamic equilibrium with tetramers. The tetramer, less likely to form aggregate, must be first disrupted into monomer to further misfold. Toxic oligomers were also reported as being “on-pathway” or “off-pathway” to amyloid fibril formation. Many factors, such as the posttranscriptional modification and SNCA mutations in A53T and E46K promote to form pathological oligomers, presently considered to be the most toxic structure of α-syn, which is further folded to form amyloid fibril (rich in β-sheet structure), the accumulation of which leads to the formation of intracellular inclusions called Lewy Body. **(b)** Established interactions between α-syn and cellular components. The misfolded α-syn can be degraded by UPS and ALP. Certain oligomeric species present toxicity via interactions with cellular components by mechanisms that include: (1) alteration of cytoskeletal integrity; (2) membrane disruption and pore formation; (3) nuclear dysfunction; (4) inhibition of vesicle docking; (5) UPS dysfunction; (6) ALP impairment; (7) reduction of mitochondrial activity; and (8) chronic ER stress. UPS, ubiquitin-proteasomal system; ALP, autophagy-lysosomal pathway; ER, endoplasmic reticulum.

Which physiological state is the major component of α-syn? Which aggregate species is the most toxic to neurons? In aqueous solution, α-syn has no defined structure and is now widely referred to as a natively unfolded protein. However, α-syn is prone to form oligomeric or fibrillar structures either in the cytoplasm or in association with the cellular membrane, via a hydrophobic NAC region responsible for the aggregation and becomes insoluble in certain pathological conditions (Figure 1a). Alpha-synuclein oligomers were reported recently to represent a continuum of species ranging from unstable low-molecular weight particles to mature fibrils via stable elongated oligomers composed of more than 15 α-syn monomers that possess seeding capacity (Pieri et al., 2016). Oligomers have been also reported as being “on-pathway” or “off-pathway” to amyloid fibril formation. Covalent bonding by oxidative modifications may be involved in stabilizing “off-pathway” oligomers (Roberts and Brown, 2015). In addition, recent results suggest that endogenous α-syn, under physiological conditions, exists predominantly as a structured and helical tetramer that must firstly be disrupted into monomers to misfold further (Bartels et al., 2011). To investigate on this confusion, nuclear magnetic resonance and electron paramagnetic resonance spectroscopy were used to acquire atomic resolution insights into the native structure of intracellular α-syn (Theillet et al., 2016). This study expounded a predominant monomeric state of α-syn in the cytoplasm, but the tetrameric state has not yet been completely ruled out. The tetramer hypothesis, which proposes that α-syn could exist in a dynamic equilibrium between monomeric and tetrameric states, could be accepted as up to 20% of α-syn exists in a tetrameric state at anytime (Alderson and Bax, 2016). An alternative view demonstrates α-syn existing as tetramers in normal, but at a decreased tetramer and monomer ratio in genetic mouse models of familial PD (Dettmer et al., 2015). The destabilization of physiological tetramers by missense mutations causing PD and the neurotoxicity and inclusions induced by markedly decreasing tetramers suggest that decreased α-helical tetramers and increased unfolded monomers facilitate subsequent pathological aggregation of monomers into disease-mediating assemblies (Dettmer et al., 2015). Similarly, a dynamic equilibrium was also demonstrated to exist between monomeric cytoplasmic α-syn and multimeric membrane-bound α-syn (Burre et al., 2013). Within the cells, α-syn normally adopts α-helical conformation. Under certain circumstances, however, this protein undergoes a profound conformational transition to a β-sheet-rich structure that polymerizes to form toxic oligomers and amyloid plaques. Different types of oligomers, which were generated either by polymerization of monomers or sonication of fibrils, were both found to have a negative impact on neuronal excitability, as indicated by patch-clamp recordings of pyramidal neurons in neocortical brain slices from mouse (Kaufmann et al., 2016). Tissues that preincubated with α-syn oligomers, but not monomeric or fibrillar species, displayed an increase in synaptic transmission (Diogenes et al., 2012). This supports the emerging notion that α-syn oligomer (profibril), rather than α-syn fibril, seems to be neurotoxic (Winner et al., 2011; Cremades et al., 2012; Forloni et al., 2016). Following lentiviral injection into the SN area of rat, researchers observed that the oligomer-forming mutants caused the most severe dopaminergic loss. Similarly, the α-syn mutants, which promote the formation of oligomer and reduce the propensity of fibrillization, cause increasing neurotoxicity in worms, flies, and mammalian neurons. Furthermore, purified oligomeric species can induce cellular reactive oxygen species (ROS) (Chen et al., 2015). Furthermore, the mature fibrils exhibit attenuated toxicity to human neuronal model cells and seeding activity (Lam et al., 2016). In brief, the oligomerization, but not the fibrillization, of α-syn may cause the selective vulnerability of dopamine neurons loss and accelerate progression of the disease. Some experimental results, however, are strongly conflicting with this widely accepted paradigm. The pathophysiological effects of α-syn oligomer, fibrils, and ribbons were assessed by injecting different assemblies into the SN of rats with or without α-syn overexpression (Peelaerts et al., 2015). In terms of neurotoxicity, fibrils caused neurodegeneration and behavioral defects, but the highest burden of LBs, LNs, and glial cytoplasmic inclusions were observed after inoculation with ribbons (Peelaerts et al., 2015). In other studies, however, fibrils appeared to exhibit higher toxicity (Bousset et al., 2013; Pieri et al., 2016). It is worth noting that α-syn oligomers spread more efficiently than fibrils and ribbons, and this raises the question regarding spread of α-syn (Peelaerts et al., 2015). Whether oligomers or fibrils are more toxic to neurons is a subject of intense debate and further studies.

### Pathways Implicated in Toxicity of α-Syn to Neurons

**FIGURE 2:**
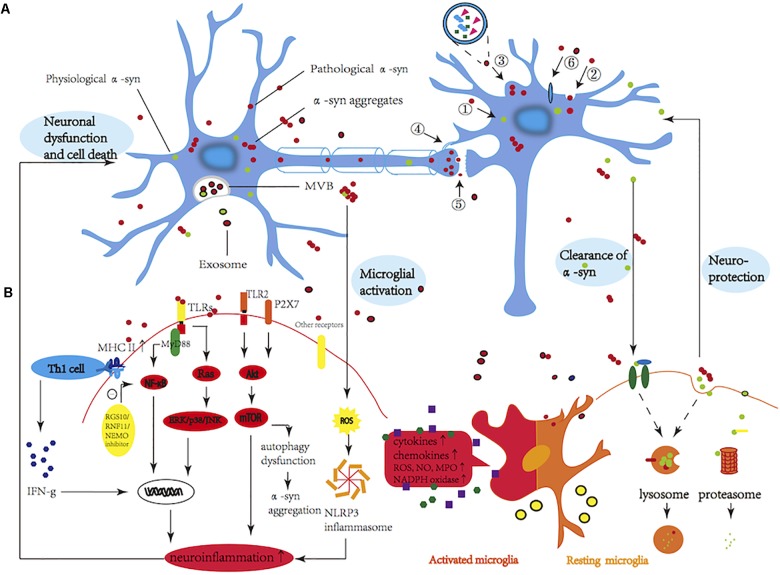
**(A)** Potential mechanisms involved in propagation of α-syn. Spreading mechanisms of α-syn in neighboring cells are multiple and can occur via (1) passive transmission through membrane fusion; (2) classical exocytosis and endocytosis; (3) packaged-exosomes; (4) tunneling nanotubes (a direct connection between two cells); (5) axonal transport and transsynaptic junction; and (6) receptor-mediated internalization. **(B)** Molecules and signaling pathways involved in α-syn-mediated microglial activation. Excessive microglial activation can increase the production of pro-inflammatory cytokines (TNF-α, IL-1β, IL-6, and INF-γ), and induce an oxidative stress response, including the release of reactive oxygen species (ROS) and nitric oxide (NO) as well as the production of NADPH oxidase. Toll-like receptors (TLRs) play a vital role in recognizing pathogen-associated molecular patterns (PAMPs) and initiating innate immune responses via distinct signaling pathways, including NF-κB and MAPK activation. Activation of TLR2 resulted in the accumulation of α-syn as a result of the inhibition of autophagic activity through regulation of the AKT/mTOR pathway. Other receptors that are involved in the α-syn-induced microglial response include FcγRs/CD36/P2 × 7R/EP2/Mac-1/Ion channels. Also, α-syn induced the expression of matrix metalloproteinases (MMPs) and stimulated the activities of MAPK, NF-κB, and AP-1. In addition, MMPs may activate microglial protease-activated receptor-1 (PAR-1) in an autocrine or paracrine manner and increase microglial inflammatory signals (not shown in the diagram). Furthermore, major histocompatibility complex II (MHC-II) and Th1 cells were targeted recently for the activation of microglia. Exosomes are specifically and efficiently taken up by microglia via a macropinocytotic mechanism and are released via activation of 5-hydroxytryptamine (5-HT2a, 2b, and 5-HT4) receptors. Activated exosomes expressed a high level of MHC–II, which may be a potentially important pathway for the activation of microglia. In contrast, regulator of G-protein signaling 10 (RGS10), RING finger protein 11 (RNF11), and NF-κB essential modulator (NEMO) inhibitors exert negative regulation on NF-κB signaling, producing a dampened immune response. Finally, microglial cells are also able to phagocytose different forms of extracellular α-syn, via ubiquitin-proteasomal system (UPS) and autophagy-lysosomal pathway (ALP), presenting a mechanism of clearance that might be even beneficial for neuronal survival. The CD36 (a scavenger receptor), FcγRs (Fc gamma receptors), Mac-1 (macrophage antigen-1 receptor), EP2 (prostaglandin E2 receptor subtype 2), P2 × 7R (purinergic receptor P2×, ligand-gated ion channel 7), and plasma membrane ion channels.

Alpha-synuclein is able to transit between multiple different conformations. Prefibrillar α-syn has been proposed to contribute to neurodegeneration by perturbing cellular ion homeostasis via calcium influx, seeding the assembly of soluble α-syn into higher molecular weight aggregates, and/or disequilibrating cellular proteostasis. In addition, α-syn was reported recently to inhibit specifically signaling of the brain-derived neurotrophic factor/tropomyosin-related kinases B, leading to dopaminergic neuronal death (Kang et al., 2017). However, the mechanism of how these α-syn species injure neurons is yet undefined. The following mechanisms may engage in and play an essential role in the progress of the disease (Figure 1b). (1) Oxidative stress: increased intracellular level of ROS may in part be a consequence of α-syn oligomers disturbing mitochondrial respiration and uncoupling oxidative phosphorylation (Nakamura, 2013). In addition, upregulated generation of cytosolic ROS in response to α-syn oligomers may originate partly from NADPH oxidase, a superoxide-generating enzyme (Choi et al., 2012). Surrounding activated glia also contribute to neuronal oxidative stress by upregulating NADPH oxidase and iNOS expression and releasing superoxide and nitric oxide into the extracellular space. Furthermore, copper bound to α-syn oligomers can catalyze ROS formation and has revealed the multifaceted role of the α-syn-Cu(2^+^) complex in oxidative stress-associated oligomer toxicity in cells and *in vitro* (Wang C. et al., 2010). (2) Membrane disruption and pore formation: oligomers may either insert into membranes forming porelike structures that could act as non-selective channels, resulting in abnormal calcium influx (or other ions), or their interaction with the membrane may disturb the lipid packing, giving rise to membrane defects (Tsigelny et al., 2012). This hypothesis is supported by the cryo-EM of annular oligomers in membranes (Zhang et al., 2013), single-channel electrophysiology that appears to show discrete stepwise changes consistent with pore opening and closing, and evidence that oligomer-induced permeability is inhibited by both anti-aggregation compounds (Schmidt et al., 2012) and the oligomer-specific A11 antibody (Yoshiike et al., 2007). In addition, α-syn oligomers were observed also to cause an enhanced lipid flip-flop with a fast membrane permeabilization in a fraction of the large unilamellar vesicles (Stockl et al., 2012). Depleting the calcium in the extracellular space reduced the oligomer-induced cell death, further highlighting the importance of membrane health and calcium homeostasis (Angelova et al., 2016). (3) Mitochondrial dysfunction: soluble α-syn oligomers recapitulate several mitochondrial phenotypes, alter membrane potential, disrupt Ca^2+^ homeostasis, and enhance cytochrome *c* release (Luth et al., 2014; Reeve et al., 2015). (i) Toxic species impair mitochondrial structure and complex I activity as well as mitochondrial dynamics and mitophagy; (ii) the α-syn associates to the mitochondrial inner and outer membrane; (iii) accumulation of intramitochondrial ROS and Ca^2+^influx leads to reduction in mitochondrial membrane potential and opening of “mitochondrial permeability transition pores” (mPTP); (iv) release of cytochrome *c* leads to activation of caspase-3 and caspase-9 and further initiation of apoptosis leading to cell death; (v) the α-syn binds to mitochondrial chaperone mortalin, voltage-dependent anion-selective channel protein 1, and translocase of outer mitochondrial membrane (Betzer et al., 2015) and interacts with the F-type ATPase (Ludtmann et al., 2016); (vi) the α-syn overexpression, in particular A53T mutant, results in an increase in mitophagy (autophagy of mitochondria) and further leads to a drastic reduction in the number and size of mitochondria, a process for which Parkin gene is essential (Choubey et al., 2011). (4) Endoplasmic reticulum (ER) stress: cellular accumulation of deformed α-syn associates with the ER membrane causes morphologic dysfunction such as dilated cisternae, increases the level of ER chaperones, and disrupts ER-Golgi vesicular transport, all of which result in toxic ER stress. Moreover, A53Tα-syn has been shown *in vitro* to inhibit the formation of the ER/Golgi SNARE quaternary complexes, which involves the assembly of the a4-helix bundle, important for vesicle docking and fusion. (5) Mechanism of protein degradation: accumulation of α-syn reduces the efficiency of clearance of specific protein substrates, thereby, interfering with the cellular physiology, and eventually leading to cell injury. Individuals with a heterozygous mutation in the lysosomal hydrolase, glucosidase 1 (GBA1), have approximately a 7% probability of developing sporadic PD (Sidransky et al., 2009). Proteasome activity seems to be restored by the addition of antibodies that neutralize the interaction or disrupting α-syn oligomers pharmacologically with Congo Red, which preferentially binds and disturbs β-sheet structure (Xilouri et al., 2013b). Inhibition of lysosomal and autophagosomal fusion with bafilomycin led to an increase in exosomal α-syn, while reduction was shown with rapamycin (Danzer et al., 2012). In addition, non-aggregated α-syn, particularly with A30P or A53T mutations, has the ability to impair the lysosome-associated membrane protein type 2A (LAMP-2A)-mediated uptake of chaperone-mediated autophagy (CMA) substrates into lysosomes (Cuervo et al., 2004). The compensatory increase in macroautophagy that follows CMA-blockade may be partly responsible for cell death. (6) Altered cytoskeleton formation: The study that reduced tubulin polymerization led to changes in cytoskeletal integrity has been undertaken in dopaminergic neurons of mice, where recombinant soluble oligomers were applied. Furthermore, α-syn oligomers were reported to bind preferentially to cytoskeletal proteins, such as the microtubule-associated protein dynamin-2 (Betzer et al., 2015). Mild overexpression of an oligomerizing E57K α-syn variant impaired microtubule stability and reduced neurite complexity (Prots et al., 2013). When expressed in a transgenic mouse model, the E57K oligomer-forming variant of α-syn was associated with dendritic and synaptic loss and reduced levels of synapsin 1 and synaptic vesicles (Rockenstein et al., 2014). Finally, α-syn oligomers, which decreased axonal transport by impairing microtubule stability and interaction between kinesin and microtubules (Prots et al., 2013; Cartelli et al., 2016), prevented fusion of synaptic vesicle and the formation of SNARE complex (Choi et al., 2013). (7) Neuroinflammation (Figure 2B): in cellular assays, either via being secreted into the extracellular space or by causing neuronal death, misfolded α-syn species can trigger activation of glia (Beraud et al., 2011), one of the hallmarks of the neuroinflammatory process, from which released factors could create oxidative stress, enhance protein misfolding, and promote further inflammation and degeneration of dopaminergic cells (Gao et al., 2011). Astrocytes are able to take up and degrade α-syn oligomers via the lysosomal pathway (Lindstrom et al., 2017). Kim et al. demonstrated that α-syn oligomers led to inflammatory responses of the microglial via activation of TLR2 (Kim et al., 2013), but the interaction was highly conformation-selective; only certain types of oligomers activated TLR2, whereas monomers, fibrils, and some oligomer types did not. In addition, activation of the β1-integrin receptor by neuron-released α-syn is responsible for the morphological changes and migration of microglia. Moreover, microglia isolated from adult, but not young mice, displayed reduced phagocytosis of exosome-associated α-syn oligomers and enhanced the secretion of TNF-α (Bliederhaeuser et al., 2016). (8) Nuclear dysfunction: the targeting of α-syn to the nucleus has been proposed in many aspects. (i) Alpha-synuclein binds directly to histones, reduces the level of acetylated histone H3 in cultured cells, and inhibits acetylation in histone acetyltransferase assays (Kontopoulos et al., 2006); (ii) G51D (a novel mutation in the α-syn gene), enriched in the nuclear compartment and hyperphosphorylated at S129, was observed to exacerbate α-syn-induced mitochondrial fragmentation (Fares et al., 2014); (iii) overexpression of transcription factor EB (TFEB), a major transcriptional regulator of the autophagy-lysosome pathway (ALP), reduces the clearance of α-syn oligomers (Decressac et al., 2013); (iv) suppression of peroxisome proliferator-activated receptor γ coactivator 1α (PGC-1α) results in the downregulation of nuclear-encoded subunits of the mitochondrial respiratory chain and increases the loss of dopaminergic neuron (Eschbach et al., 2015); (v) increased calcineurin-dependent nuclear import of the nuclear factor of activated T cells (CN/NFATc3 signaling pathway) contributes to α-syn-mediated loss of midbrain dopaminergic neurons (Luo et al., 2014).

## Seeding and Propagation Properties

### Cell-to-Cell Transmission of α-Syn

Based on the presence of pathological α-syn aggregates in different regions of the brain, a seminal study by Braak et al. (2003) suggests that Lewy pathology evolves in a sequential and predictable fashion, beginning in the olfactory system, peripheral autonomic nervous system, and dorsal motor nucleus of the glossopharyngeal and vagal nerves (stage 1) and extending to develop in the medulla oblongata and the pontine tegmentum (stage 2) and later in the dopamine neurons of the SNpc (stage 3). The pathology then worsens, and the α-syn inclusions reach the temporal cortex (stage 4) and lastly affect the neocortex (stages 5 and 6) (Braak et al., 2003). The dorsal motor nucleus of the vagal nerve may be critical in inducing the development of pathology in rostral locations of the brain. However, the mechanisms underlying the progress of the disease remain unclear. The disease could begin in the gut and move retrogradely to the brain via the vagal nerve or it could start in the vagal dorsal motor nucleus and move to the spinal cord and gut in an anterograde fashion. Furthermore, it could also begin in the periphery at multiple autonomic sites and subsequently transmit to the spinal cord. Following injections of adeno-associated viral (AAV)-overexpressed α-syn into midbrains of rats, Ulusoy et al. (2017) found the presence of human α-syn in vagal motor neurons and gastric nerve endings of visceromotor vagal projections. In addition, α-syn also efficiently transferred between neurons and glia (Lee H.J. et al., 2010; Kovacs et al., 2014; Loria et al., 2017). With the background that constipation and anosmia both appear in early PD, exogenous α-syn forms were used to testify the pathologic spread from the gastrointestinal tract or the olfactory bulb to the brain in mice (Rey et al., 2013; Holmqvist et al., 2014). The vagal route of α-syn retrograde transport is observed not only by systemic administration of environmental toxin, rotenone, but also by injecting AAV overexpressing human α-syn into the vagal nerve (Pan-Montojo et al., 2012; Ulusoy et al., 2013). Specifically, the notion has been strongly proposed that α-syn may self-propagate and spread progressively between interconnected brain regions via a cell-to-cell transmission mechanism (Figure 2A). This hypothesis started gaining significant traction following the pathological analyses of postmortem brain tissue from patients with PD, who received fetal mesencephalic tissue grafts into the striatum. In 2008, two postmortem studies reported that grafted nigral neurons developed pathological α-syn or LBs similar to those observed in the brain of host, after implantation of healthy embryonic mesencephalic dopaminergic neurons into the striatum of patients with PD (Kordower et al., 2008; Li et al., 2008). Host-to-graft propagation of α-syn-positive Lewy-like pathology clearly revolutionized the understanding of the kind of disorders that PD is. Numerous experimental studies (*in vivo* and *in vitro*) have shown strong evidence for prion-like transmission of α-syn (Volpicelli-Daley et al., 2011; Luk et al., 2012; Mougenot et al., 2012; Masuda-Suzukake et al., 2013, 2014; Paumier et al., 2015; Prusiner et al., 2015; Woerman et al., 2015; Abdelmotilib et al., 2017; Koller et al., 2017). Coculturing two cell lines expressing different fluorescent tagged-α-syn resulted in progressive emergence of double-labeled cells (Hansen et al., 2011), and treating tagged α-syn cells with misfolded α-syn tagged with a different probe leads to significant colocalization in cultured cells (Bousset et al., 2013). This hypothesis was supported further by *in vivo* studies. Through injecting synthetic α-syn fibrils into striatum of non-transgenic mice, 6 months later, researchers expounded “cell-to-cell transmission” of pathologic α-syn and Parkinson-like Lewy pathology in anatomically linked regions of the striatum and the development of lesions in motor dysfunction and neurodegeneration (Luk et al., 2012; Mougenot et al., 2012). Consistently, intracerebral injections of synthetic α-syn fibrils or homogenates of brain derived from α-syn-transgenic mice exhibiting Lewy pathology into young asymptomatic α-syn-transgenic mice stimulate both the formation of Lewy body like inclusions and the onset of motor signs (Masuda-Suzukake et al., 2013). Similar observations are found following the intramuscular or intravenous injection of α-syn seeds, adding a new piece of evidence for the spread theory of α-syn (Peelaerts et al., 2015). Non-human primates inoculated with nigral LB-enriched fractions containing pathological α-syn from patients with PD are subject to gradual neuronal degeneration (Recasens et al., 2014). The cell-to-cell transmission of pathologic α-syn and Lewy pathology in anatomically interconnected regions indicates that α-syn fibrils have prion-like properties. However, a recent study showed that the pattern and extent of dissemination of α-syn pathology did not necessarily follow the expected neuroanatomic connectivity (Sorrentino et al., 2017). Further, unlike the M83^+/+^ (A53T) mice that develop extensive α-syn pathology following the injection with recombinant fibrillar αsyn, in M47^+/+^ (E46K) mice, the α-syn deposition is largely restricted to the injection site with no evidence for the spread (Sacino et al., 2014). It is also worth noting that the Mendez et al. team reported no Lewy pathology in surviving grafts that included dopamine and serotonin neurons after postmortem analyses of five patients with PD 9–14 years after the transplantation of fetal midbrain cell suspensions (Mendez et al., 2008). Although the evidence for prion-like propagation of α-syn is extensive, variation exists and may be associated with differences in the incubation period, pattern of α-syn pathology, the graft environment, the years postgrafting, animal models used, and individual differences between patients with PD.

### Potential Mechanisms Involved in α-Syn Propagation

An outstanding issue to be solved relates to the factors initiating the transfer and spread of α-syn. Several hypotheses attempt to address the cellular mechanisms underlying progressive α-syn transmission spreading throughout different regions of brain (Figure 2A). The passive release of α-syn into the surrounding extracellular milieu might be a logical possibility. However, only monomeric α-syn can pass the cell membrane by diffusion process, which relies on a so-far unidentified membrane translocator, suggesting that physiological formation of multimers prevent nascent α-syn from passively exiting the cell. Furthermore, a lack of increase in cerebrospinal fluid (CSF) α-syn levels between diseased and control individuals limits the likelihood of this process as the main mechanism. In addition, the presence of intact neural connection is reported as a prerequisite for the propagation of pathology, as neuronal injury and degeneration did not exacerbate interneuronal α-syn transfer (Ulusoy et al., 2015). A small portion of cellular α-syn has been present in the lumen of vesicles, of which α-syn proteins were secreted from neuronal cells through unconventional exocytosis, which collectively refers to endoplasmic reticulum/Golgi-independent exocytosis. Vesicular α-syn is more prone to aggregate than cytosolic α-syn. It is hypothesized that cells have a mechanism to recognize misfolded α-syn and selectively translocate such proteins into vesicles, reducing intracellular neurotoxicity (Lee et al., 2014). Furthermore, grafted neurons could take up this released α-syn through endocytosis and membrane receptors. Alpha 3-subunit of Na^+^/K^+^-ATPase has been reported to be a cell surface partner of α-syn assemblies (Shrivastava et al., 2015). Lymphocyte-activation gene 3 (LAG 3) (Mao et al., 2016) and neurexin 1a (Shrivastava et al., 2015) were identified recently as receptors for preformed fibrils (PFFs), but not monomers, initiating transmission of α-syn and accelerating their spread throughout the brains of mice. Other putative mechanisms involved in cell-to-cell propagation pathways, such as transmission through direct penetration, axonal transport (Freundt et al., 2012), or via *trans*-synaptic means (Masuda-Suzukake et al., 2014), have also been proposed. In addition, the role of exosome in spreading of α-syn pathology is gaining attention (Guo et al., 2016; Wang et al., 2017), and it was recently shown that the exosome isolated from the plasma of patients with PD contain higher levels of α-syn when compared to ones from control individuals (Shi et al., 2014), suggesting potential contribution of exosome to the spread of toxic agents. Recently, a study showed that α-syn oligomers were found in the exosomal fraction of primary neurons or cell lines (Danzer et al., 2012). The explanation could be that oligomerization is an important determinant of exosomal release. Exosome-associated α-syn oligomers are more prone to internalize and be toxic compared with exosome-free α-syn oligomers (Danzer et al., 2012). The aggregation of exogenous α-syn can be accelerated by exosomes, caused by the exosome lipid (Grey et al., 2015). Moreover, CSF exosomes derived from PD can also initiate oligomerization of soluble α-syn in target cells in a dose-dependent manner and confer disease pathology (Stuendl et al., 2016), supporting the notion that exosomes contain α-syn “seeds” or “strains” that spread α-syn aggregates in the brain. In contrast to CSF exosomal α-syn levels, which are reported to be lower in PD group than healthy control, the concentrations of plasma α-syn in L1CAM-containing exosomes are significantly higher in patients with PD (Shi et al., 2014; Stuendl et al., 2016). Plasma exosomal α-syn is likely to be CNS derived, implying an increased rate of transport from the CNS to the blood in patients with PD (Shi et al., 2014). Notably, PD-linked human PARK9/ATP13A2 is involved in exosomal biogenesis and α-syn secretion (Park et al., 2014; Tsunemi et al., 2014). The surviving DA neurons in the substantia nigra of patients with PD express higher levels of ATP13A2 mRNA and protein than controls (Ramirez et al., 2006; Ramonet et al., 2012). Inhibitory effects of ATP13A2 on the α-syn toxicity were shown later in primary DA neurons (Gitler et al., 2009). The deficiency of ATP13A2 was reported further to lead to decreased secretion of α-syn in exosomes, which, in turn, contributes to α-syn accumulation (Kong et al., 2014; Tsunemi and Krainc, 2014). Altogether, ATP13A2-mediated α-syn release via exosomes may indicate a potential neuroprotective role of exosomes in PD. Leucine-rich repeat kinase 2 (LRRK2) is also involved in the secretion of exosomes because of the colocalization of multivesicular bodies (MVBs) and LRRK2 (Alegre-Abarrategui et al., 2009). The PD-linked mutations in LRRK2 induce the formation of skeinlike abnormal MVBs and the release of exosomes. Notably, autophagic inactivation and lysosomal dysfunction were found to cause a dramatic change in exosomal α-syn release into the cell medium (Alvarez-Erviti et al., 2011; Danzer et al., 2012). The exosome-mediated release of α-syn clears toxic proteins and provides a partial compensation (Danzer et al., 2012). *In vitro* study, an observed acceleration of α-syn aggregation in the presence of exosomes might be caused by the ganglioside lipid composition of exosomes, thereby, providing further evidence that exosomes contribute to the nucleation of α-syn aggregation (Grey et al., 2015). It is also possible that exosomal encapsulation of α-syn might confer partial protection against degradation of extracellular protein. Thus, secreted exosomes are proposed to be a mechanism for the removal of unnecessary α-syn from neurons, but exosomal hypersecretion also serves as a potentially pivotal player in the propagation of α-syn seed in the brain, just as the “Trojan horse” hypothesis of exosomes in neurodegeneration, a mechanism leading to the death of cells by shipping of toxic agents in the exosomes from cell-to-cell (Ghidoni et al., 2008). The precise role of exosomes in the spreading of α-syn pathology needs to be further detailed.

## Innate and Adaptive Immunity and α-Syn

The description for the involvement of inflammatory response proceeded from the observation of activated microglia of postmortem brain regions of patients with PD. The degree of microglial activation was assessed by staining for CD68 and human leukocyte antigen-DR (HLA-DR) and this suggests that α-syn may activate the innate immune system directly (Croisier et al., 2005). A postmortem analysis of the brain revealed that there is a 10-fold greater infiltration of CD4+ and CD8+ T-lymphocytes into the SNpc of patients with PD, compared with age-matched controls (Brochard et al., 2009). In the MPTP model of PD, adoptive transfer of T-cells from mice that have been immunized to the nitrated C-terminus of α-syn to mice administered MPTP leads to increased neurodegeneration (Benner et al., 2008). Both pro-inflammatory Th1 and Th17 subtypes were found to enhance the neurodegeneration in response to MPTP, with Th2 cells having no effect (Reynolds et al., 2010). Additionally, adoptive transfer of a Treg-enriched population protected the mice from neurodegeneration in response to MPTP (Reynolds et al., 2010). Furthermore, in a passive transfer study into Rag1^−/−^ mice, CD4+ T-cells acted in a FasL-dependent, IFN-γ-independent manner to mediate MPTP toxicity to dopaminergic neurons, while CD4^−/−^ animals, but not CD8a^−/−^, showed attenuation of neurodegeneration (Brochard et al., 2009). However, microglia may mediate their dopaminergic neurotoxicity via IFN-γ (Mount et al., 2007). In mice, given a single intraperitoneal injection of a subneurotoxic dose of MPTP, knocking out IFN-γ was sufficient to ameliorate completely microglial activation (Barcia et al., 2012). Recently, a work by Cebrian et al. (2014) has shown that treatment of primary microglia with α-syn led to increased microglial expression of IFN-γ. In this study, expression of IFN-γ induced expression of surface MHC-I and processing of antigen in catecholaminergic neurons, allowing them to be targeted selectively for degeneration *in vitro* by CD8+ T-cells. The B-cells have not been observed yet in the brains of patients with PD patient; however, immunoglobulin G (IgG), but not IgM, is deposited in the SNpc on dopaminergic neurons in postmortem brains of patients with PD, colocalizing with α-syn aggregates (Orr et al., 2005).

## Microglia-Mediated Inflammation and α-Syn

The accumulation of α-syn leads to the activation of microglia and fosters neuroinflammation, which as potential contributing factors facilitate the progressive degeneration of dopaminergic neurons (Figure 2B). However, the exact mode by which α-syn and microglia affect each other is still poorly understood. The study demonstrating that microglia are recruited to the site of α-syn aggregation raises the possibility that α-syn may be a chemoattractant to inflammatory response (Wang et al., 2015). Alpha-synuclein may induce also the migration of BV-2 microglial cells by the upregulation of CD44 and membrane type-1 matrix metalloproteinase (MT1-MMP) (Kim et al., 2009). *In vivo*, imagings of widespread activation of microglia by the peripheral benzodiazepine receptor-binding radioligand [11C]-(R) PK11195 PET in patients with PD have been accepted as an impactful measurement of microglial activation (Gerhard et al., 2006). It is conceivable that inflammation-induced changes in microglia impair both their efficacy to take up extracellular α-syn and their ability to degrade it. Recent evidence in animal models and cell cultures has confirmed the role of α-syn in initiating the activation of microglia and in inducing pro-inflammatory pathways (Fellner et al., 2013; Kim et al., 2013; Sanchez-Guajardo et al., 2013; Zhang et al., 2017). The presence of neuromelanin inside activated microglia indicates that diseased dopaminergic neurons in SN are likely being phagocytosed, thereby, allowing pathogenic α-syn to undergo processing and potential antigen presentation (Depboylu et al., 2011). Early microglia-mediated neuroinflammation was observed to be involved in initiation and progression of disease (Su et al., 2009). Similarly, PD-associated mutant forms of α-syn (A30P, E46K, and A53T) induced microglial activation, with subsequent secretion of pro-inflammatory cytokines (IL-6, IL-1β, and TNF-α) and anti-inflammatory cytokine IL-10 as well as chemokines (Jiang et al., 2015; Hoenen et al., 2016). The cytokine-transforming growth factor-β (TGF-β), known to modulate the activation of microglia, has been also observed in brains and CSF of patients with PD. Similar results were demonstrated using a BV2 line in which neuron-derived wild type and mutant α-syn were observed to increase the production of the pro-inflammatory cytokines, TNF-α, and IL-1β (Rojanathammanee et al., 2011). Moreover, components of the complement system, C3d, C4d, C7, and C9, have been found in association with degenerating neurons and α-syn inclusions in brains of patients with PD (Yamada et al., 1992), and in the case of C1q, in association with activated microglia surrounding degenerating neurons (Depboylu et al., 2011). Lastly, oxidative stress plays essential roles in the toxicity of activated microglia via increased production of NO and ROS. As a part of the feedback circle in the pathological process of PD, neuroinflammation and oxidative stress affect and potentiate each other to promote dopaminergic cell death (Zhang et al., 2017). Although microglia take up a significant amount of aggregated α-syn (i.e., α-syn oligomers) for subsequent degradation, their degradative capacity becomes overwhelmed, resulting in limited clearance of α-syn and its associated toxic cellular effects. As a consequence, the dead dopaminergic cells release excessive α-syn, leading to further activation of inflammatory response and oxidative stress in microglia vice versa more neurodegeneration. However, a recent study (Koller et al., 2017) that injected mouse wild-type α-syn fibrils into the hippocampus of AAV-IL-6-expressing mice demonstrated a result of widespread gliosis and concurrently reduced α-syn inclusion pathology, supporting a beneficial role of inflammatory priming of the CNS in wild-type mice challenged with exogenous α-syn. In addition, silencing endogenous α-syn results in a similar pattern of nigral degeneration observed following overexpression of α-syn, initiating a neuronal-mediated neuroinflammatory cascade (Benskey et al., 2018). Therefore, the relationship between α-syn and microglia needs further more investigations.

### Receptors Activated by α-Syn in Microglial Activation

Several different receptor systems have been implicated in the uptake of α-syn into microglia. In addition to Fcγ receptor (FcγR)-mediated phagocytosis, toll-like receptors (TLRs) were demonstrated to play a crucial role in the recognition, internalization, and activation of microglial cells (Figure 2B). Particularly, the pattern-recognition receptors, TLR2 and TLR4, were found to be essential regarding α-syn phagocytosis and α-syn-dependent activation (Fellner et al., 2013; Kim et al., 2013). TLRs initiate innate immune responses via nuclear factor-kappa B (NF-κB) and mitogen-activated protein kinase (MAPK) signal pathways. Preconditioning of microglia with α-syn strongly affects the responses induced by TLRs stimulation, especially TLR2/1- and TLR7-mediated responses, through augmenting secretion of IL-6 and chemokine (Roodveldt et al., 2013). Furthermore, major histocompatibility complex II (MHC-II) was targeted recently for the activation of microglia (Harms et al., 2013). The subchronic AAV-mediated overexpression of α-syn leads to MHC-II-dependent microglial activation, inflammatory cytokine release, and subsequent degeneration of dopaminergic neurons. Data in support of this idea suggests that PD-linked mutations in HLA-DR (a component of MHC-II) lead to the presence of DR-positive reactive microglia (Hamza et al., 2010). Moreover, extracellular aggregated α-syn was phagocytosed and further targeted to the light chain 3B (LC3B+) autophagosome for degradation in microglia (Cao et al., 2012). Additionally, FcγR^−/−^mice are protected from neuroinflammation after overexpression of AAV-mediated α-syn (Cao et al., 2010), suggesting a role of FcγR in mediating the internalization of extracellular aggregated α-syn and engaging in downstream NF-κB-dependent signaling cascades (Cao et al., 2012). In addition, EP2 (prostaglandin E2 receptor subtype 2) may be involved in α-syn-induced microglial activation, as ablation of EP2 enhances significantly the microglia-mediated clearance of α-syn aggregates in MPTP-treated mice (Jin et al., 2007). Notably, Lee E.J. et al. (2010) undertook a study to determine the roles of MMPs in α-syn-induced microglial activation and found that MMP-8 inhibitor suppressed TNF-α production more efficaciously than did MMP-3 or MMP-9 inhibitors. Further investigation of the role of PAR-1 in α-syn-induced inflammatory reactions indicated that PAR-1-specific inhibitor and PAR-1 antagonist suppressed significantly cytokine levels and NO and ROS production in α-syn-treated microglia. However, these studies identified different α-syn conformations as the primary ligand for microglial activation as well as distinct receptors to bind. These inconsistent results may indicate a non-specific nature of α-syn-induced microgliosis or perhaps a capacity for multiple receptors to sense changes in levels of extracellular α-syn, regardless of the conformation.

### Relations Between Exosomal Signaling and Microglial Activation

Research on the exosomal signaling in the CNS is scant, especially, with regard to microglial cells. Neuron-derived exosomes were found to transfer α-syn toxic forms between neuronal and non-neuronal cells (such as microglia) thereby, contributing to PD spreading. Exosomes are specifically and efficiently taken up by microglia via a macropinocytotic mechanism without inducing a concomitant inflammatory response and are preferentially internalized in microglia that seem to have no antigen-presenting capacity (Fitzner et al., 2011). Recently, a study demonstrated that 5-hydroxytryptamine (5-HT) stimulates the release of exosomes from microglial cells via activation of 5-HT2a, 5-HT2b, and 5-HT4 receptors (Glebov et al., 2015). Moreover, the Wnt3a signal in microglia, through a glycogen synthase kinase 3 (GSK3)-independent mechanism, was also reported to result in exosomal release (Hooper et al., 2012). However, to date, how these signal pathways play a role in dopaminergic degeneration is still lacking. In an AAV-based model, a study demonstrated that microglia spread tau via exosomal secretion, and inhibiting exosomal synthesis and depletion of microglia dramatically suppressed tau propagation *in vitro* and *in vivo* (Asai et al., 2015). A similar result was reported that exosomes derived from α-syn-treated microglia showed a marked increase, suggesting that the exosomal secretion by microglia is stimulated by extracellular α-syn aggregation. These activated exosomes expressed a high level of MHC-II and membrane TNF-α and induced neuron cells apoptosis (Chang et al., 2013). As mentioned earlier, the pathogenic species of α-syn could activate microglia leading to more exosomal secretion, induce spread to healthy neurons, and trigger more aggregation of α-syn in recipient neurons. Additionally, an increased release of inflammatory exosomes has been demonstrated in α-syn-activated microglia, suggesting the exosomal role in initiating inflammatory cascade (Bliederhaeuser et al., 2016). Unexpectedly, recent studies found that exosomal uptake by neuron was less efficient than microglia (Fitzner et al., 2011; Zhuang et al., 2011; Fruhbeis et al., 2013). Thus, microglia may prevent pathology by inducing clearance of α-syn-containing exosomes as well as may result in the propagation of misfolded α-syn and the spread of inflammation. Microglia isolated from adult mice, in contrast to that from young ones, display phagocytosis deficits of free and exosome-associated α-syn oligomers combined with enhanced TNF-α secretion (Bliederhaeuser et al., 2016), putting emphasis on the switching of microglia from neuroprotective effects in the young brains to neurotoxic roles in the aged ones.

## Targeting α-Syn for Pd Treatment

**FIGURE 3:**
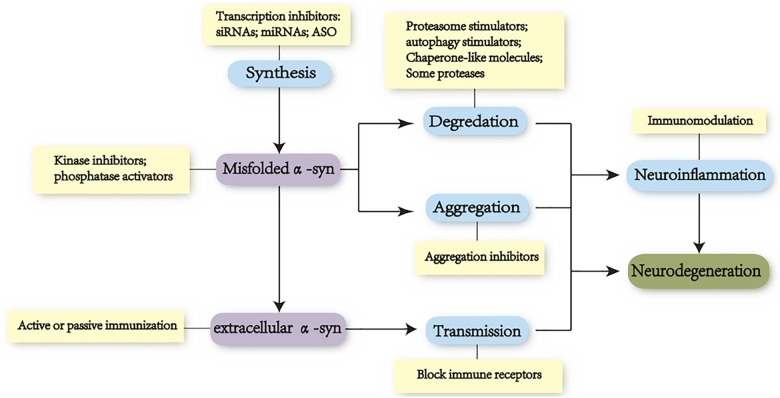
Focus on toxicity of α-syn as a therapeutic target. Toxicity of alpha-synuclein to neurodegeneration is associated tightly with the dynamic equilibrium of the protein synthesis, aggregation, and clearance. Levels of specific conformations (oligomers and protofibrils) vary in different stages of PD. Disease-modifying therapeutic strategies are mainly focused on these processes as well as inhibiting cell-to-cell propagation: (i) reducing α-syn synthesis with small interfering RNA (siRNA), microRNA (miRNA), small hairpin RNA (shRNA), and transcription inhibitors; (ii) increasing degradation of α-syn via UPS and ALP; (iii) reducing aggregation of α-syn via heat-shock proteins (hsp40/70/104), aggregation inhibitors, antioxidant, and posttranslational modification approaches (oxidation, nitration, phosphorylation, and C-terminal cleavage); (iv) blocking the propagation of α-syn with immunotherapies by targeting extracellular α-syn or exosome and by blocking putative receptors in recipient cells; and (v) seeking neuroprotective strategies including anti-inflammation and antioxidant.

No treatment is, to date, available to cure PD. Studies that may help prevent progression of disease have great potential to be the much-required medical treatment. increasing evidence from both *in vitro* and *in vivo* studies has supported the vital role of α-syn in progression of PD. Therefore, just as landmark discoveries that identified PD as a disease of dopamine deficiency led to the development of dopamine replacement therapy with levodopa, so will the understanding of pathological mechanisms of PD, spurred on by the study of α-syn, lead to experimental therapies targeting α-syn, especially the oligomeric species (Winner et al., 2011; Cremades et al., 2012). The progressive misfolding and accumulation of α-syn have been recently related to an imbalance in levels of the α-syn synthesis, aggregation, and clearance. Hence, α-syn-directed therapeutics might require alleviating the neurotoxic gain of α-syn via many ways: reduction of α-syn synthesis, inhibition of α-syn aggregation, and an increase of α-syn clearance (Figure 3).

### Methods to Reduce Synthesis of α-Syn

Decreasing production of α-syn at a time prior to aggregation might prevent its aggregation and rescue the function and viability of neuronal populations that are vulnerable in PD. The evidence that direct infusion of small interfering RNA (siRNA) reduced hippocampal and cortical α-syn expression, and that systemic exosomal siRNA decreased aggregation of α-syn and reduced synthesis of α-syn via microRNAs (miRNAs), siRNAs, antisense oligonucleotides (ASO), or other transcription inhibitors may be of potential therapeutic interest (Lewis et al., 2008; Cooper et al., 2014). Following unilateral siRNA infusions, infusion against α-syn in squirrel monkeys, levels of α-syn were reduced by 40–50% relative to the untreated side (McCormack et al., 2010). Moreover, Burton and coworkers inhibited 35% expression of endogenous α-syn in the SN of adult rat by AAV-mediated delivery of a short hairpin RNA (shRNA) targeting the endogenous rat SNCA transcript (Zharikov et al., 2015). However, controversies were reported regarding the potential neurotoxicity caused by extensive loss of endogenous α-syn. A recent study using siRNA revealed that reduction of α-syn resulted in a pronounced amphetamine-induced behavioral asymmetry consistent with the progression of disease (Gorbatyuk et al., 2010). Similarly, knockdown of endogenous α-syn in dopamine neurons of non-human primates reproduces the pattern of nigrostriatal degeneration characteristic of PD (Collier et al., 2016). Following a unilateral injection of AAV expressing an siRNA targeting endogenous α-syn into the SNpc of adult rats, another study reported a rapid upregulation of MHC-1 with 50% loss of nigrostriatal neurons in the SNpc and a corresponding loss of nigrostriatal terminals and dopamine concentrations within the striatum (Benskey et al., 2018). However, coexpression of both rat α-syn and α-syn siRNA partially reversed the neurodegenerative and behavioral effects (Gorbatyuk et al., 2010). Thus, how therapies administered systemically might even gain greater access to reducing α-syn in peripheral tissues is not yet well understood. A safe degree of reduction would have to be established prior to clinical trials. In addition, β2-adrenoreceptor (β2AR) was identified as a regulator to modify the transcription of the SNCA gene. Mechanistically, β2AR ligands were shown to modulate SNCA transcription through histone H3 lysine 27 acetylation of its promoters and enhancers. Treatment with the β2AR agonist, salbutamol, reduced expression of α-syn in the substantia nigra of wild-type mice and protected TH-neurons from MPTP toxicity in mice. Conversely, its antagonist propranolol was associated with an increased risk to PD (Mittal et al., 2017).

### Methods to Increase Clearance of α-Syn

Extracellular α-syn was cleared through proteolysis by extracellular proteases and cell-mediated uptake and degradation by the cells of brain parenchyma, of which microglia are the principal scavengers of extracellular α-syn aggregates. Specific targeting of extracellular α-syn will not interfere with the normal function of intracellular α-syn and would be an additional advantage as a therapeutic strategy. The therapeutic possibility to alleviate the endocellular α-syn burden is to promote the activity of the ubiquitin-proteasome system (UPS) and autophagy-lysosomal pathway (ALP) (Wong and Cuervo, 2010; Fowler and Moussa, 2018). Activity of selected HSPs (Opattova et al., 2015) and stimulation of ubiquitination (Prabha et al., 2012) may result in an increased level of specific substrates available for the degradation in UPS, by which the shift of misfolded proteins could be prevented from the initial conversion to toxic molecules. Recently, a study presented the retention in endoplasmic reticulum 1 (RER1), as a mediator in UPS-dependent degradation of α-syn, interacting with both cytosolic and ER/Golgi transmembrane proteins (Park et al., 2017). Nigral injection of a proteasomal inhibitor, lactacystin, induced a PD-like motor phenotype, emphasizing on the role of proteasome in degradation of α-syn in pathology of PD (Savolainen et al., 2017). The UPS seems to degrade α-syn independent of the preexisting α-syn burden, whereas macroautophagy appears to occur primarily when α-syn levels are abnormally increased (Ebrahimi-Fakhari et al., 2012). The α-syn expression is found to increase with a marked concomitant elevation of LAMP-2A, overexpression of which leads to upregulation of CMA in PD models (Mak et al., 2010; Xilouri et al., 2016). Several studies have confirmed the neuroprotective role of stimulation of autophagy with overexpression of transcription factors (Malagelada et al., 2010; Decressac et al., 2013; Xilouri et al., 2013a). Rapamycin [mammalian target of rapamycin (mTOR)-dependent pathway] or trehalose (mTOR-independent enhancers of autophagy activation) may represent potential approaches to increase the function of autophagy and result in an increased clearance of protein aggregates (Malagelada et al., 2010; Bove et al., 2011; Moors et al., 2017). Another strategy to achieve inhibition of mTOR works by reducing pyruvate transport into mitochondria using a modulator of the mitochondrial pyruvate carrier (MPC) called MSDC-0160, which enhances the autophagy in α-syn-induced toxicity in *C. elegans* (Ghosh et al., 2016). However, poor specificity (effects on other essential pathways) and many downstream factors involved in the mTOR biology might, in turn, restrict its potential clinical application (Bove et al., 2011). On the contrary, better understanding of autophagic gene expression, such as beclin1 and TFEB, may present promise as an avenue for future therapy (Spencer et al., 2009; Decressac et al., 2013). Neuropathological analysis revealed that a lentivirus-expressing beclin1 injection ameliorated the synaptic and dendritic pathology, with reduced accumulation of α-syn through an enhanced lysosomal activation and reduced alterations in the autophagy pathway (Spencer et al., 2009). Similarly, the function of lysosome was strengthened by overexpression of TFEB, which afforded robust neuroprotection via the clearance of α-syn oligomers and was aggravated by microRNA-128-mediated repression of TFEB, inducing neurodegeneration and progression of further disease (Decressac et al., 2013). Moreover, several proteases such as neurosin, matrix metalloproteinases (MMPs), calpain, cathepsin D, and plasmin have also been proposed to cleave α-syn aggregation (Park and Kim, 2013; Xilouri et al., 2013b). Chaperone like molecules, including HSPs and β-syn, promote the binding of α-syn, leading to decreased extracellular oligomers (Auluck and Bonini, 2002). Finally, small-molecule chaperones that would drive correct folding of the mutant lysosomal enzyme, β-glucocerebrosidase (GCase), which was encoded by GBA1 gene and identified as the most-common genetic risk factor for PD, have been reported to increase GCase activity, contributing to degradation of α-syn. Ambroxol, an FDA-approved mucolytic, acts as a chaperone and improves lysosomal function in cells with GBA mutations *in vitro* (McNeill et al., 2014) and increases GCase activity in non-human primates *in vivo* (Migdalska-Richards et al., 2017). Two separate phase 2 trials to test safety, tolerability, and efficacy of Ambroxol in PD are under the way (ClinicalTrials.gov Identifier: NCT02941822; NCT02914366). There is also preliminary evidence that poly(DL-lactide-co-glycolide) (PLGA) acidic nanoparticles (aNP) restore impaired lysosomal function in a series of PD models, adding to the range of therapeutic options of promoting the degradation of α-syn (Bourdenx et al., 2016). Finally, many drugs used in oncology have presented recently their efficacy in PD. For example, nilotinib is an approved drug for chronic myelogenous leukemia and an inhibitor of c-abl activity. This is enhanced in the brain tissue of patients with PD and leads to a downstream increase in α-syn phosphorylation and aggregation as well as a reduced function of PD (Lonskaya et al., 2014). It has been shown to attenuate α-syn levels in A53T transgenic mice and protects SNpc dopamine neurons from toxicity (Hebron et al., 2013).

### Methods to Reduce Aggregation of α-Syn

Additionally, identifying the factors that reduce aggregation of α-syn may arise as potential targets for therapeutic interventions. Application of sonicated α-syn fibrils was found to induce robust aggregation and spreading of neuronal endogenous α-syn, either by culturing with primary neuron or by injection into non-transgenic brains of mice, supporting the hypothesis that the exogenous aggregates directly seed the aggregation of native proteins. However, whether endogenous α-syn can induce similar responses in neurons remains unclear. A direct approach to blocking aggregation of α-syn is to stabilize the native α-syn conformation or inhibit the transmission of α-syn oligomers. Mannitol, a blood-brain barrier (BBB) disrupter, was reported to inhibit aggregation of α-syn into fibrils, with low concentration *in vitro* (Shaltiel-Karyo et al., 2013). In addition, brain-permeable small molecules that upregulate the level or activity of several molecular chaperones, particularly the HSP70 and HSP104, reduced aggregation of α-syn and protected against α-syn toxicity, notably, through a decrease in oligomer concentrations (Auluck and Bonini, 2002; Lo Bianco et al., 2008). The oligomer modulator anle138b was tested to be neuroprotective (Wagner et al., 2013). Moreover, many new aggregation inhibitors such as the prolyl oligopeptidase inhibitor KYP-2047, curcumin, “Molecular Tweezer” CLR01, and fibril-conversion epigallocatechin-3-gallate (EGCG) were also investigated and found with neuroprotective properties (Pandey et al., 2008; Bieschke et al., 2010; Wang M.S. et al., 2010; Myohanen et al., 2012; Prabhudesai et al., 2012; Attar and Bitan, 2014; Savolainen et al., 2014). The SH-SY5Y cells overexpressing the A53T mutant α-syn and treated with curcumin showed a reduction in aggregated A53T α-syn, whereas there was a significant increase in destabilized PFFs and toxicity, suggesting the formation of α-syn toxic species after fibrillar dissociation (Pandey et al., 2008; Wang M.S. et al., 2010). The CLR01 is proved to inhibit the aggregation of α-syn in a zebrafish model expressing human wild-type α-syn (Prabhudesai et al., 2012). The NPT200-11 and NPT088 are two candidates that are currently in clinical testing phase. The NPT088, a fusion protein combining human immunoglobulin backbone as well as a general amyloid interaction motif (or GAIM) was reported to bind α-syn aggregates and reduced the accumulation of proteinase K-resistant protein (Krishnan et al., 2014). Another approach is to reduce the posttranslational modifications of α-syn (Lee et al., 2011). The mutation of Ser129 in the prevention of phosphorylation completely suppresses dopaminergic neuronal loss, in contrast to the results that phosphorylation of Ser129, via the G protein-coupled receptor kinase 2 (Gprk2), significantly enhances α-syn toxicity (Chen and Feany, 2005). The overexpression of polo-like kinase 2 (PLK2) to phosphorylate α-syn at Ser129 was shown to be neuroprotective (Oueslati et al., 2013). Moreover, mice deficient in GM1 ganglioside increased aggregation of α-syn that can be partially attenuated by treatment with a membrane-permeable analog of GM1 ganglioside (Wu et al., 2011), and this has been examined by a single-center, delayed-start RCT with positive results (Schneider et al., 2013). Recent studies suggest that therapeutic strategies directed at the prevention of α-syn-mediated microglial activation, either by blocking direct interaction with surface receptors on microglia or interrupting the microglial-mediated inflammation cascades, may prevent downstream pathogenesis (Joers et al., 2017).

### Immunotherapies Against α-Syn

The discovery of lymphatic vessels in the brain highlights a role for the immune system in the brain. Immunotherapies including active and passive immunization have presented neuroprotective effects (Masliah et al., 2011; Bae et al., 2012; Valera and Masliah, 2013). The accumulation of α-syn emerged as a promising therapeutic target for immunotherapies (Masliah et al., 2011; Bae et al., 2012; Games et al., 2014; Mandler et al., 2014; Tran et al., 2014; Schneeberger et al., 2016). Alpha-synuclein transgenic (α-syn-tg) mice that were vaccinated with recombinant human α-syn produced high relative affinity antibodies, which ameliorated α-syn-related pathology in neuronal cell bodies and synapses. Other active immunization approaches using AFFITOPEs (AFFiRiS AG), which contain short peptide fragments that mimic the abnormal conformations of C-terminus of human α-syn, have been studied in animal models. Vaccination with one of these AFFITOPEs (AFF 1) resulted in high antibody titers against aggregates of α-syn and decreased accumulation of α-syn oligomers (Mandler et al., 2014). Despite the promising preclinical data, active immunization was considered to be associated in some cases with vasculitis and autoimmune responses (Menendez-Gonzalez et al., 2011). Notwithstanding these risks, active vaccination has theoretical advantages in that its long-lasting immune responses might also be advantageous and it might lead to high antibody levels. The immunogen used to generate the therapeutic antibody in the first-published passive immunization study was a monoclonal antibody (9E4) against the C-terminus of α-syn (Masliah et al., 2011). This promotes clearance of α-syn via the lysosome/autophagosomes pathway within neurons. Monoclonal antibodies directed against the C-terminus of α-syn in mThy1-α-syn tg mice model reduced brain levels of C-terminus-truncated α-syn in axons, and ameliorated its propagation and PD-like pathology (Games et al., 2014). Another approach that used antibodies to target the N-terminal or central region of α-syn has also reduced α-syn-induced nigral cell death and the number of activated microglia (Shahaduzzaman et al., 2015). By a recombinant single-chain antibody variable targeting α-syn oligomers, under the promoter for platelet-derived growth factor-β, the protein conjugate decreased neuropathology and neuronal loss of α-syn (Spencer et al., 2014). Moreover, antibody mAb47, designed to recognize α-syn oligomers and treat the oligomeric load of Thy1-hA30P transgenic mice, showed a reduction in soluble and membrane-associated α-syn and an improvement in their motor phenotype (Lindstrom et al., 2014). In addition, two α-syn antibodies (PRX002 and BIIB054) have progressed to clinical trials (Bergstrom et al., 2016; Schneeberger et al., 2016). The PRX002 was tested in a first-in-human, placebo-controlled, phase 1 trial in PD with up to 96.5% reduction of unbound serum α-syn levels, where it appeared safe and suppressed α-syn levels in the blood (Schenk et al., 2017). The phase 2 study, an year-long efficacy study in 300 patients with PD, is now in process, with the latest update that single and multiple doses of PRX002 were generally safe and well tolerated and resulted in robust binding of peripheral α-syn and dose-dependent increases of PRX002 in CSF, reaching concentrations that may be expected to engage extracellular aggregated α-syn in the brain (Patel et al., 2018). Similar results were reported for BIIB054 (Alzforum, 2017). A study from Lee’s group treated hippocampal neurons with different α-syn monoclonal antibodies [mAbs (Syn211 and Syn303)] 30 min before transduction with hWTα-syn PFFs and demonstrated that this reduced formation of LBs/LNs and rescued synapse/neuron loss by preventing both uptake of PFFs and subsequent templated propagation and cell-to-cell transmission of α-syn pathology (Tran et al., 2014). The BAN0805 is another clinical-phase antibody that targets oligomeric forms of α-syn that are thought to be pathogenic (Fagerqvist et al., 2013). Indeed, a majority of the added PFFs remained outside neurons, where they colocalized with the α-syn mAbs, but not with intracellular neuronal markers. Additionally, generation of conformation-specific antibodies for the “pathogenic” forms of α-syn would avoid interfering with the physiological function of α-syn. Finally, granulocyte-macrophage colony-stimulating factor (GM-CSF) induces tolerogenic dendritic cells (DCs) that increase Treg numbers and activities, which serve to attenuate proinflammatory responses and protect nigrostriatal dopaminergic neurons (Schutt et al., 2018). Recently, an RCT study demonstrated that sargramostim showed modest improvement in UPDRS-III scores, which paralleled improved magneto encephalography-recorded cortical motor activities and regulatory T-cell (Treg) numbers and function (Gendelman et al., 2017).

### Strategies Targeting Propagation of α-Syn

Prion-like propagation of α-syn largely strengthened the potential efficacy of drugs such as antibodies that are tricky to get into cells. Given that exosomes are able to penetrate the BBB and are involved in transfer of α-syn from cell-to-cell, it would be efficacious for exosomes to be engineered to deliver therapeutic drugs/antibodies to target the cells. To achieve widespread delivery of siRNAs to the brain, a new drug delivery system was proposed, consisting of modified exosomes incorporating α-syn siRNA (Cooper et al., 2014), which was found to reduce α-syn messenger RNA (mRNA) and protein levels in α-syn-tg mouse model. Furthermore, antibodies against α-syn specifically target and aid in the clearance of extracellular α-syn proteins by microglia, through the FcγR, thereby, preventing the neuron-to-astroglia transmission and their actions on neighboring cells (Bae et al., 2012). Moreover, the use of intrabodies that are specifically built to target intracellular aggregates of α-syn would be more fascinating and efficacious (Dehay et al., 2015). To block immune receptors for extracellular α-syn in accepted cells seems to be another way to regulate transmission of α-syn. Cell-surface LAG3/CD233 protein, a neuronal receptor, is involved in the endocytosis of aggregated α-syn (Mao et al., 2016). The LAG3-blocking antibodies significantly decrease misfolded α-syn toxicity and transmission *in vitro* (Anderson et al., 2016; Mao et al., 2016). Additionally, blocking α-syn exocytosis was once deemed as an effective approach, but this process was limited by intracellular accumulation of misfolded and aggregated α-syn. It is also worth noting that a third type of immunotherapy based on cellular-mediated immunity, which involves the activation of phagocytes, natural killer cells, antigen-specific cytotoxic T lymphocytes and the release of various cytokines in response to an antigen has been explored for the potential treatment of α-synucleinopathies (Reynolds et al., 2010). Though α-syn immunotherapy has become a widespread therapy for PD, many questions remain to be unraveled. How can different conformational antibodies recognize the specific structure of the peptide? How do they target intracellular α-syn or extracellular α-syn? Is α-syn immunotherapy a promising enough concentration to the antibody for PD treatment? How to prevent adverse effects or autoimmune inflammatory responses? Thus, further more investigations are warranted to detect the mechanism of how immunotherapy targets and halts cell-to-cell transmission of α-syn pathology, as well as the safety and productiveness of the process. Finally, 14-3-3 proteins were reported recently to reduce the cell-to-cell transfer and propagation of pathogenic α-syn (Wang et al., 2018) and, therefore, antibodies or drugs used to enhance the protein’s activity may reduce the transmission of α-syn.

### Challenges Facing Clinical Trials With α-Syn Therapies

Though many programs have entered clinical testing, different challenges targeting clinical trials with therapies that target α-syn are still difficult to handle. Firstly, due to the nature of α-syn as an intrinsically disordered protein with extreme conformational diversity, it is difficult to associate a particular structural species to neuronal toxicity, and it remains unclear as to what molecular species of α-syn is the best target for modifying therapy. In addition, the approach that reduces levels of α-syn may present a potential benefit to symptomatic release by reducing dopaminergic neuron death. Furthermore, a confounding challenge we are facing now is the possibility that α-syn may adopt different conformations depending on its environment and vary from patient to patient (Peng et al., 2018). Undoubtedly, this increases the difficulty of treating PD, especially, in the aspects of disease-modifying therapies. Thus, the development of effective and sensitive α-syn biomarkers is urgent for early diagnosis and assessment of clinical trials. Assays to measure total levels of α-syn in easily accessible fluids such as CSF, blood, and saliva, the putative pathological forms such as phosphorylated (pS129) and oligomeric α-syn species, and its ability to seed and nucleate further aggregation have been developed recently (Majbour et al., 2016; Shahnawaz et al., 2017). According to the Braak staging that α-syn originates from peripheral nerves, it would be a possibility to intervene in the premotor phase, such as olfactory hypothyroidism and constipation, to delay α-syn entering the CNS and slowdown the progress of PD. In addition, lessons learnt from clinical trials for Alzheimer’s disease suggest that it will likely be both costly and a prolonged follow-up time to assess the treatment effect.

## Conclusion

Mounting evidence has demonstrated the concept that α-syn may be responsible for initiating and spreading the pathological process in PD. This “prion-like hypothesis” postulates α-syn as a prion-like pathological agent and suggests that native α-syn undergoes a conformational change, promoting a α-syn misfolding, and polymerizes to form toxic oligomers. Research into the role of α-syn in the pathogenesis of PD has also transformed from the focus of protein inclusions to oligomers. Recent discoveries suggest that a variety of possible mechanisms are involved in conferring oligomeric toxicity. The accumulation of toxic proteins and mitochondrial dysfunction, particularly at axon terminals, ultimately might overwhelm the capacity of intracellular protein-degradation mechanisms. Neuroinflammation, from which released factors could enhance oxidative stress, protein misfolding, and aggregation, creates a positive feedback loop promoting the degeneration of dopaminergic cells. As a key player in disease pathogenesis, increased therapies targeting α-syn have attracted potential interest. These possibilities include treatments designed to block the synthesis, release, or uptake of the pathogenic protein by siRNA, increases its extracellular clearance by microglia, inhibit protein assembly by small molecules such as HSP, and increase degradation of aggregates by proteasome and lysosomes. Targeting the extracellular phase of transmission of α-syn in a prion-like fashion, immunotherapy might hold the key to be one of the disease-modifying strategies for slowing down of the progression of disease. However, identifying the correct species of α-syn for intervention would be important for these treatments. Consequently, further research into basic α-syn pathobiology and mechanisms, and underlying factors that influence the synthesis, aggregation, spread, and the clearance of α-syn are required, in order to provide new insights that can identify the development of refined therapeutic strategies for PD.

## Author Contributions

The review was designed by GZ and YX. Related articles were researched by KM, XG, FW, LK, SY, CH, LL, JH, and NX. The manuscript of this review was prepared by GZ and YX. TW critically revised the draft before submission.

## Conflict of Interest Statement

The authors declare that the research was conducted in the absence of any commercial or financial relationships that could be construed as a potential conflict of interest.
